# Changes in ADMA/DDAH Pathway after Hepatic Ischemia/Reperfusion Injury in Rats: The Role of Bile

**DOI:** 10.1155/2014/627434

**Published:** 2014-08-27

**Authors:** Andrea Ferrigno, Vittoria Rizzo, Alberto Bianchi, Laura G. Di Pasqua, Clarissa Berardo, Plinio Richelmi, Mariapia Vairetti

**Affiliations:** ^1^Department of Internal Medicine and Therapeutics, University of Pavia, 27100 Pavia, Italy; ^2^Department of Molecular Medicine, Fondazione IRCCS Policlinico S. Matteo, 27100 Pavia, Italy

## Abstract

We investigated the effects of hepatic ischemia/reperfusion (I/R) injury on asymmetric dimethylarginine (ADMA, a nitric oxide synthase inhibitor), protein methyltransferase (PRMT) and dimethylarginine dimethylaminohydrolase (DDAH) (involved, resp., in ADMA synthesis and degradation), and the cationic transporter (CAT). Male Wistar rats were subjected to 30 or 60 min hepatic ischemia followed by 60 min reperfusion. ADMA levels in serum and bile were determined. Tissue ADMA, DDAH activity, DDAH-1 and CAT-2 protein, DDAH-1 and PRMT-1 mRNA expression, GSH/GSSG, ROS production, and lipid peroxidation were detected. ADMA was found in bile. I/R increased serum and bile ADMA levels while an intracellular decrease was detected after 60 min ischemia. Decreased DDAH activity, mRNA, and protein expression were observed at the end of reperfusion. No significant difference was observed in GSH/GSSG, ROS, lipid peroxidation, and CAT-2; a decrease in PRMT-1 mRNA expression was found after I/R. Liver is responsible for the biliary excretion of ADMA, as documented here for the first time, and I/R injury is associated with an oxidative stress-independent alteration in DDAH activity. These data are a step forward in the understanding of the pathways that regulate serum, tissue, and biliary levels of ADMA in which DDAH enzyme plays a crucial role.

## 1. Introduction

Nitric oxide (NO) is abundantly synthesized from the amino acid arginine, by the action of NO-synthase (NOS), a family of enzymes with an endothelial, neuronal, and inducible isoform [1]. Asymmetric dimethylarginine (ADMA) is an endogenous inhibitor of these enzymes because it competes with L-arginine for each of the three isoforms of NOS: it is considered an important marker of endothelial dysfunction because of its inhibiting role in NO synthesis. An increase in ADMA leads to vasoconstriction, increases platelet aggregation, increases cell adhesion to the endothelium, and increases vascular muscle cell proliferation [2]. The first step in the synthesis of methylarginines is the methylation of protein arginine residues by intracellular enzymes termed protein methyltransferases (PRMTs). The second step relates to the proteolytic degradation of the methylated protein which produces free ADMA and symmetric dimethylarginine (SDMA), and the latter is not biologically active [3]. The liver and kidneys represent the main sites of ADMA metabolism and excretion. The kidney plays an important role in the elimination of dimethylarginine from the body, since ADMA is found in human urine [4]. An additional pathway was found for ADMA, namely, metabolic degradation by dimethylarginine dimethylaminohydrolase (DDAH), an enzyme that is widely distributed in rats and human subjects, but, in particular, in the liver, kidney, and pancreas [5, 6]. Nijveldt et al. provide a detailed insight into the liver's handling of dimethylarginine, demonstrating that it plays a crucial role in ADMA metabolism, with DDAH taking up a large amount of ADMA from the systemic circulation [7]. Over the past few years, types 1 and 2 isoforms of DDAH emerged as critical regulators of NO bioavailability [8]. Studies of gene silencing or deletion in rodents led to the conclusion that plasma levels of ADMA are regulated by DDAH-1, whereas the significance of DDAH-2 lies in preserving the endothelial function [8].

ADMA is also able to interfere with NO synthesis by competing with arginine and SDMA for cellular transport across cationic amino acid transporters (CATs). Interestingly, the liver expresses CATs abundantly, especially CAT-2A and CAT-2B, suggesting a higher uptake of ADMA in this organ as compared with the heart, lungs, and kidneys [9].

ADMA has been shown to correlate with cardiovascular risk factors [10, 11] and is considered a predictor of cardiovascular events [12]. Moreover, ADMA plasma concentration increases in patients suffering from hepatic dysfunction [13] and end-stage kidney disease [14] and in situations of endothelial dysfunction and increased atherosclerotic risk [15, 16]. Furthermore, in ischemia/reperfusion (I/R) injury, ADMA, which is increased by reducing DDAH activity, may well influence NO production by competing with arginine for the binding site in the active NOS centre [17]. Indeed, evidence for the protective effects of NO synthesis was seen when NOS inhibitors dramatically worsened liver necrosis and apoptosis [18]. Correlation between methylarginine derivatives and liver function and survival after liver transplantation was also observed [19]. However, this does not exhaust the relationship between ADMA and hepatic I/R injury. Indeed, the molecular mechanisms involved in I/R injury are not completely understood and only a few works have reported changes induced by hepatic I/R injury on the ADMA/DDAH pathway which needs to be considered as a point of interest potentially capable of reducing the effects of I/R. In addition, a previous study merely reported cirrhosis by bile duct ligature (BDL) which induced an increase in plasma ADMA levels; this did not happen with thioacetamide- (TAA-) induced cirrhosis [20]. Yet, no explanation about this event has been reported. Understanding the mechanisms involved in ADMA elimination increases the possibility of understanding its modulation which particularly crucial in several pathological conditions [10–14].

The present study was carried out to clarify whether (1) ADMA clearance also occurs through the bile and (2) hepatic I/R induces changes in ADMA levels by quantifying its content in hepatic tissue, serum, and bile samples. In addition, enzymatic activity and protein expression of DDAH and mRNA expression of DDAH-1 and PRMT-1 and CAT-2 protein at the end of reperfusion were examined.

## 2. Materials and Methods

### 2.1. Animals

The use of animals in this experimental study was approved by the National Institute for Research, and the animals were cared for in accordance with its guidelines. Male Wistar rats (200–250 gr, 2-3 months old; Charles River, Calco, Lecco, Italy) with free access to water and food were used.

### 2.2. Materials

All reagents were of the highest grade of purity available and were purchased from Sigma (Milan, Italy).

### 2.3. Ischemia/Reperfusion (I/R) Procedure

**Figure 1:**
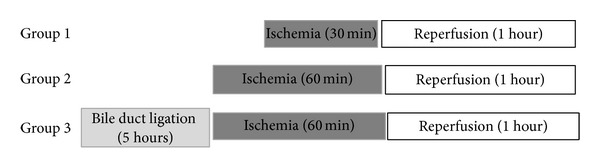
Schematic illustration of the experimental groups.

The rats were anesthetized with sodium pentobarbital (40 mg/kg i.p.), the abdomen was opened via a midline incision, and the bile duct was cannulated (PE-50). Ischemia to the left and median lobe was induced by clamping the branch of the portal vein and the branch of the proper hepatic artery after bifurcation to the right lobe for 30 or 60 min with microvascular clips [21] with the abdomen temporarily closed with a suture. After 30 or 60 min of ischemia (*n* = 8 each group), the abdomen was reopened, the clips were removed, the abdomen was closed again, and the liver was allowed to reperfuse for 60 min (Figure 1). By using partial rather than total hepatic ischemia, portal vein congestion and subsequent bacterial translocation into the portal venous blood were avoided. A set of 60 min ischemia experiments followed by 60 min reperfusion was performed after 5 hours of BDL (*n* = 6) (Figure 1). All the animals were maintained on warm support to prevent heat loss: rectal temperature was maintained at 37 ± 0.1°C. To prevent postsurgical dehydration and hypotension, 1 mL of saline was injected in the inferior vena cava. Sham-operated control animals (*n* = 8 each group) underwent similar manipulation of the liver hilum without vascular occlusion or BDL and were kept under anesthesia for an equal length of time.

### 2.4. Bile, Serum, and Tissue Sampling

Bile duct was cannulated as follows: bile duct was closed with a 4/0 silk suture ligation, placed in distal position. This causes the bile duct to swell upstream, becoming more discernible. Then, a cut was opened in the bile duct with spring scissors, and a slant-shaped polyethylene tubing was inserted. When the tubing was completely filled with bile, it was fixed with 4/0 silk suture ligation. This operation was conducted using magnifying spectacles. Bile was collected in darkened vials during the reperfusion period. Blood and tissue samples were also collected after 60 min reperfusion. Blood was drawn from the vena cava and centrifuged at 3000 g for 10 min at 4°C. Hepatic biopsies were quickly removed from the central lobe and immediately frozen in liquid nitrogen, as were bile and serum samples until they were analyzed.

### 2.5. Biochemical Assays

Liver injury was assessed by serum release of alanine transaminase (ALT), aspartate transaminase (AST), and alkaline phosphatase (ALP) by an automated Hitachi 747 analyzer (Roche/Hitachi, Indianapolis, IN, USA).

ADMA levels in serum, bile, and tissue were evaluated by direct ELISA kit according to the manufacturing procedure (Immundiagnostik AG, Germany). Quantitative analysis of ADMA was also performed in deproteinized rat bile by reversed-phase high-performance liquid chromatography (RP-HPLC) with o-phthalaldehyde/beta-mercaptoethanol (OPA/betaME) and fluorescence detection, as previously described, with some modifications [22]. The chromatography was carried out on the HPLC/HT400E system (ESSECI-Group, Como, Italy) equipment routinely used for amino acid analysis that allows automatic online mixing of all reagents for OPA derivatization. The derivatives were separated on a Teknokroma Mediterranean sea C18 column (4,6 × 150  mm; 3 *μ*m particle size) by binary gradient elution. Fluorescence was measured at an excitation and emission wavelength of 330 and 450 nm, respectively.

Proteins were measured according to Lowry's method using albumin as standard [23].

DDAH activity was evaluated using the method proposed by Tain and Baylis [24]. Tissue samples were homogenized in cold phosphate buffer 100 mM, pH 6.5; urease (100 U/mL) was added and samples were incubated at 37°C for 15 min. ADMA 1 mM in phosphate buffer was added (final ADMA concentration: 0.8 mM) and samples were incubated at 37°C for 60 min; the reaction was stopped by mixing 1 : 1 with 4% sulphosalicylic acid and samples were centrifuged for 10 min at 3000 g. Finally, the supernatants were assayed for citrulline as follows. Solution A (diacetyl monoxime 80 mM, thiosemicarbazide 2 mM) and solution B (H2PO4 3 M, H2SO4 6 M, NH4Fe(SO4)2 1.75 mM) were prepared, mixed 1 : 3, and added 1 : 1 to the samples. Samples were incubated at 60°C for 110 min and read spectrophotometrically at 528 nm against citrulline standards.

Both DDAH-1 and CAT-2 protein expressions were evaluated using Rat DDAH-1 and CAT-2 ELISA kit (Cusabio, Wuhan University Science Park, Wuhan, China).

DDAH-1 and PRMT-1 mRNA were analyzed by a real-time polymerase chain reaction (RT-PCR) (Table 1): total RNA was isolated from the liver samples with Trizol reagent in accordance with the method of Chomczynski and Mackey [25]. RNA was quantified by measuring the absorbance at 260/280 nm. cDNA was generated using the iScript cDNA Synthesis kit (BIO-RAD) following the supplier's instructions. Gene expression was analyzed using the Sso Advanced SYBR Green supermix (BIO-RAD). As regards housekeeping, gene ubiquitin C (UBC) and glyceraldehyde 3-phosphate dehydrogenase (GAPDH) were used (Table 1). DDAH-1, PMRT-1, UBC, and GAPDH gene amplification efficiency was 92.8%, 93.5%, 98.6%, and 97.4%, respectively, in a cDNA concentration range of 10–0.1 ng/*μ*L. The expression of the house-keeping gene remained constant in all the experimental groups considered. The amplification was performed through two-step cycling (95–60°C) for 40 cycles in a CFX Connect RT-PCR Detection System (BIO-RAD) following the supplier's instructions. All samples were assayed in triplicate. Gene expression was calculated using the ΔCt method. Comparison between groups was calculated using the ΔΔCt method.

The hepatic concentration of total glutathione was measured by an enzymatic method (Cayman Chemical Co., Ann Arbor, MI). Oxidized glutathione (GSSG) was determined after derivatization of reduced glutathione (GSSG) with 2-vinylpyridine. Determination of hepatic reactive oxygen species (ROS) was followed by the conversion of 2′,7′-dichlorofluorescein diacetate (H2DCFDA) to fluorescent 2′,7′-dichlorofluorescein (DCF). Tissue samples were homogenized (50 mg/mL) in Locke's buffer (120 mM NaCl, 2.5 mM KCl, 5 mM NaHCO3, 6 mM g-glucose, 1 mM CaCl2, and 10 mM HEPES, pH 7.4) and incubated for 20 min at room temperature with 10 mM H2DCFDA (Molecular Probes Inc.). Production of the fluorescent derivative DCF as a function of time (min) was measured using a microplate reader (Perkin Elmer Life Science, Monza, Italy). The extent of lipid peroxidation in terms of thiobarbituric acid reactive substances (TBARS) formation was measured using Esterbauer and Cheeseman's method [26]. TBARS concentrations were calculated using malondialdehyde (MDA) as standard.

### 2.6. Statistical Analysis

Data are presented as the mean ± S.E. Statistical analysis for multiple comparisons was performed by the one-way ANOVA test with Bonferroni's corrections.

## 3. Results 

### 3.1. Biliary, Serum, and Tissue Levels of ADMA

**Figure 2:**
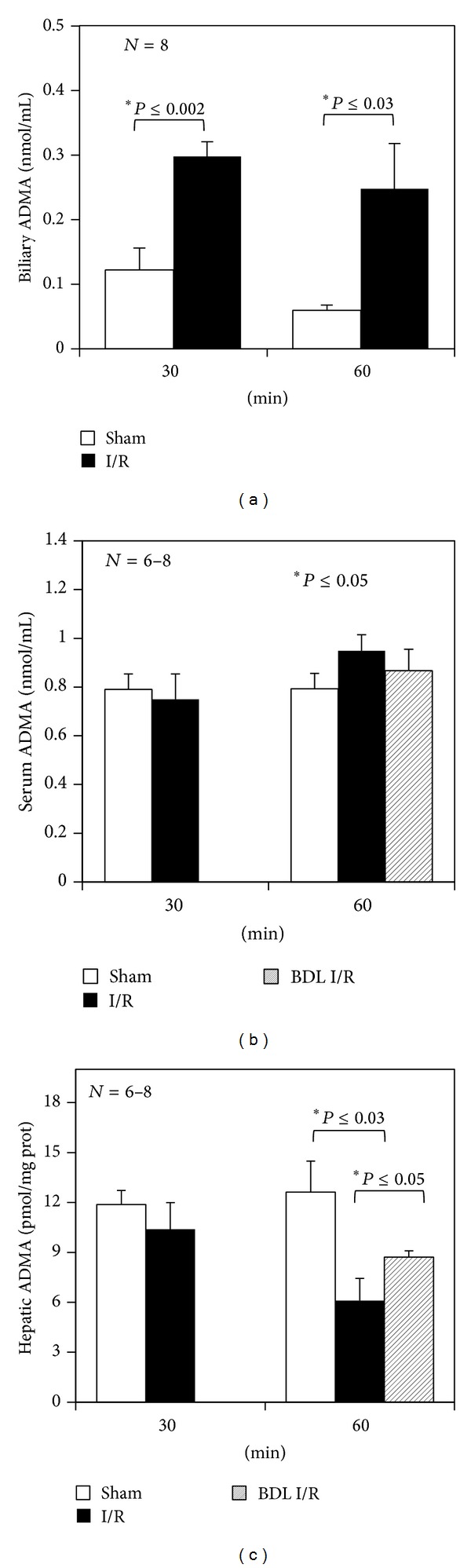
Biliary, serum, and tissue levels of ADMA at the end of reperfusion. Livers were submitted to 30 or 60 min ischemia followed by 60 min reperfusion (*n* = 8). A set of 60 min ischemia experiments followed by 60 min reperfusion was performed after 5 hours of bile duct ligation (BDL) (*n* = 6). Sham-operated control animals had similar manipulation without vascular occlusion. At the end of reperfusion, biliary, blood, and hepatic samples were collected from all groups. The results are reported as the mean ± S.E. of 6–8 different experiments.

Interestingly, ADMA was found in bile for the first time, and a time-dependent increase was observed after 30 and 60 min of ischemia (Figure 2(a)).

To confirm biliary ADMA levels, bile samples were also analyzed by the HPLC method equipped with a fluorescence detector and no significant differences when comparing data obtained by the ELISA kit were detected (i.e., 0.31 ± 0.04 versus 0.29 ± 0.02 nmol/mL, resp., in bile obtained after 30 min ischemia followed 60 min reperfusion).

The ADMA levels in the serum of control rats confirmed previously reported levels in rats [27] (Figure 2(b)). The I/R injury induced a moderate increase in serum ADMA only in 60 min ischemic rats.

Moreover, using samples obtained after 60 min of ischemia, the evaluation of tissue ADMA showed a decrease at the end of reperfusion (Figure 2(c)).

After 5 hours of bile duct ligation, no changes in serum ADMA were found (Figure 2(b)); a significant increase in intracellular ADMA was recorded when compared with the unaltered bile production I/R group (Figure 2(c)).

### 3.2. Liver I/R Injury

**Figure 3:**
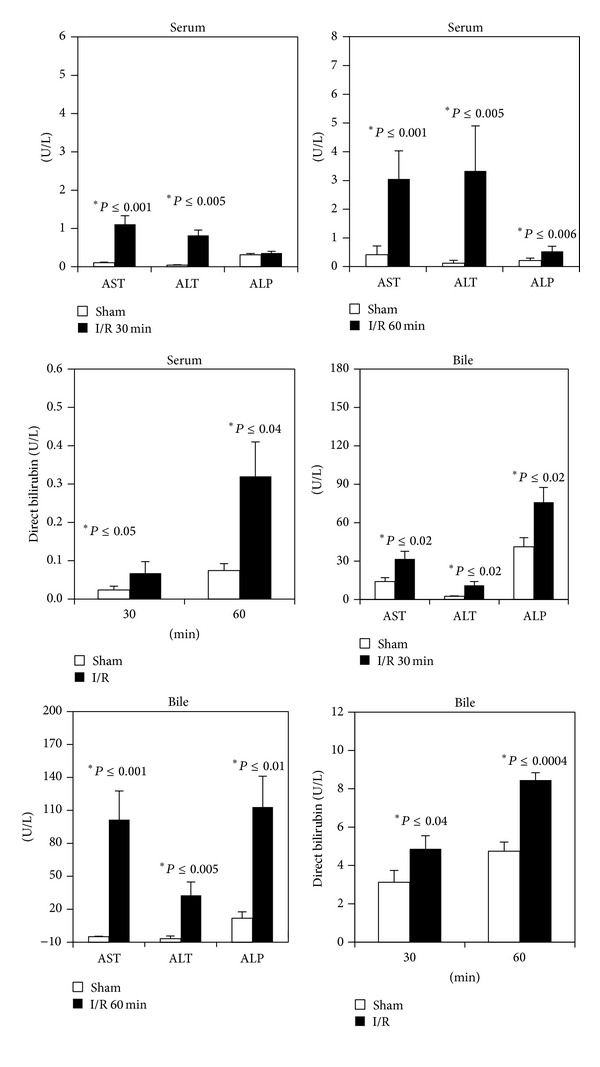
Serum and biliary levels of AST, ALT, and AP and direct bilirubin after 60 min reperfusion. Livers were submitted to 30 or 60 min ischemia followed by 60 min reperfusion. Sham-operated control animals had similar manipulation without vascular occlusion. At the end of reperfusion, blood and biliary samples were collected from all groups. The results are reported as the mean ± S.E. of 8 different experiments.

As expected, serum AST, ALT, and ALP increased in animals submitted to ischemia and reperfusion as compared with the sham-operated group (Figure 3). Liver damage depends on the ischemia period considered and a time-dependent increase was recorded: serum AST, ALT, and ALP levels increase, especially after 60 minutes of ischemia (Figure 3).

Direct bilirubin concentrations in serum were significantly higher in the I/R group as compared with the sham group. In particular, a threefold increase in serum direct bilirubin was detected in the 60 min ischemia group (Figure 3).

The analysis of bile shows a time-dependent increase in enzyme (AST, ALT, and ALP) release; high levels appeared in rats submitted to 60 min ischemia (Figure 3) particularly. The same trend was observed when comparing direct bilirubin levels in bile (Figure 3).

### 3.3. DDAH Activity, Protein, and Expression in I/R Injury

**Figure 4:**
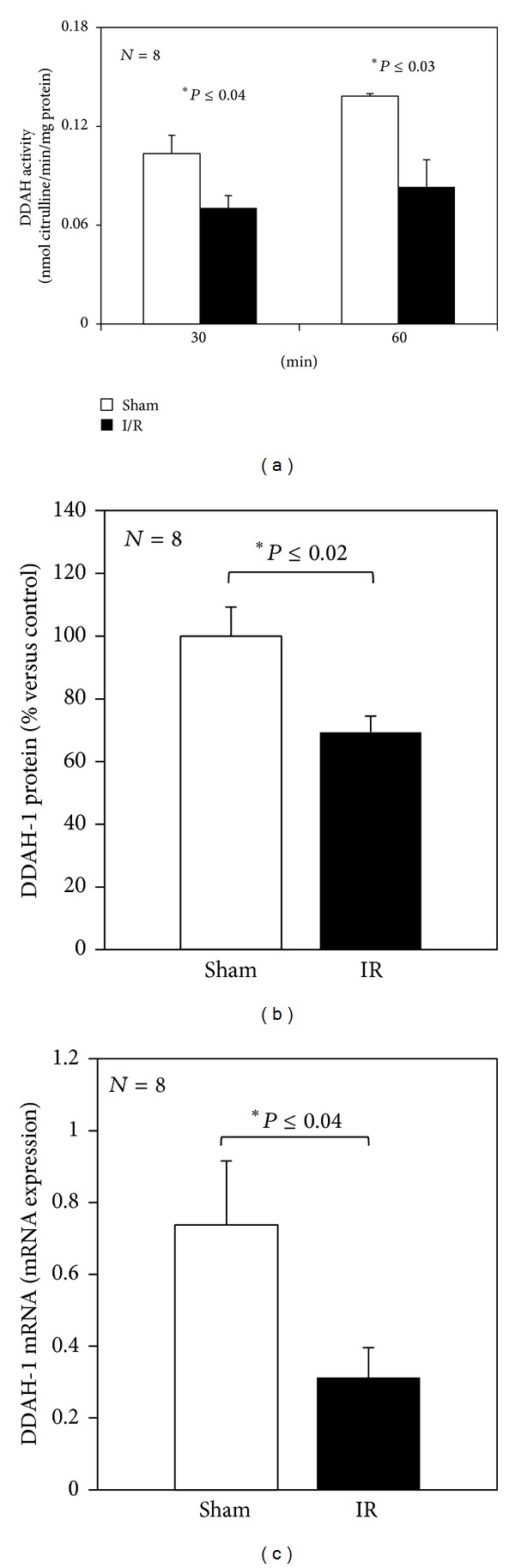
Hepatic DDAH activity and protein and mRNA expression of DDAH-1 at the end of reperfusion. Livers were submitted to 30 or 60 min ischemia followed by 60 min reperfusion, panel (a). Livers were submitted to 60 min ischemia followed by 60 min reperfusion, panels (b) and (c). Sham-operated control animals had similar manipulation without vascular occlusion. After reperfusion, liver samples were collected from all groups. The results are reported as the mean S.E. of 8 different experiments.

Decreased DDAH activity was observed at the end of reperfusion and higher reduction was found after 30 and 60 min ischemia as reported in Figure 4(a).

DDAH contains SH groups in the catalytic site and we evaluated the oxidative stress so as to provide an explanation for the reduction in its activity observed after I/R. No significant changes in GSH/GSSG ratio were observed at the end of reperfusion after both 30 and 60 min of ischemia (Table 2). The evaluation of TBARS and ROS formation showed the same trend and no significant difference in any of the experimental groups considered was found (Table 2).

Evaluation of the protein and mRNA expression of isoform DDAH-1 was performed after 60 min reperfusion in the 60 min ischemic group. The amount of DDAH-1 decrease at the end of reperfusion and the detected results were evaluated using ELISA assay (Figure 4(b)). The same was also true for the mRNA expression of DDAH-1 in the sample collected at the end of reperfusion and compared with the control group (Figure 4(c)).

### 3.4. PRMT-1 Expression in I/R Injury

**Figure 5:**
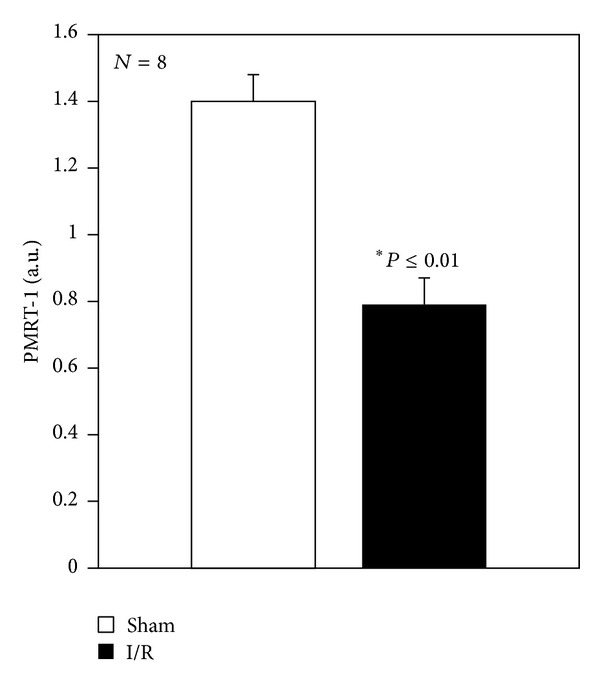
Hepatic mRNA expression of PMRT-1 at the end of reperfusion. Livers were submitted to 60 min ischemia followed by 60 min reperfusion. Sham-operated control animals had similar manipulation without vascular occlusion. After reperfusion, liver samples were collected from all groups. The results are reported as the mean S.E. of 8 different experiments.

Hepatic mRNA expression of isoform PMRT-1 was performed after 60 min reperfusion in the 60 min ischemic group. The amount of PMRT-1 decreased at the end of reperfusion as compared with the sham-operated group (Figure 5).

### 3.5. ADMA Transport via CAT-2

**Figure 6:**
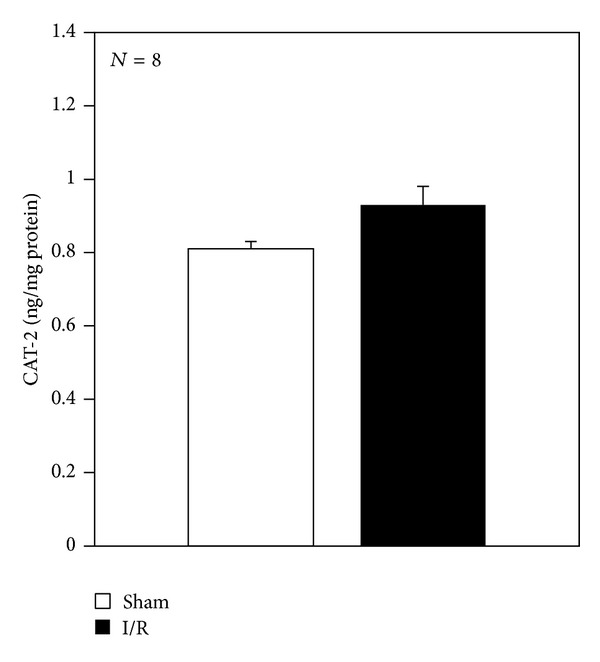
Hepatic CAT-2 protein at the end of reperfusion. Livers were submitted to 60 min ischemia followed by 60 min reperfusion. Sham-operated control animals had similar manipulation without vascular occlusion. After reperfusion, liver samples were collected from all groups. The results are reported as the mean S.E. of 8 different experiments.

No significant changes were detected for protein expression of CAT-2 transporters in rat liver samples obtained from the sham or I/R group (Figure 6).

## 4. Discussion 

This work demonstrated that the liver is responsible for the biliary excretion of ADMA, as documented here for the first time, and also that I/R injury induces changes in serum, biliary, and hepatic levels of ADMA. Notably, a significant oxidative stress-independent decrease in DDAH activity that associates with low mRNA and protein expression occurs at the end of the reperfusion period supporting the hypothesis that the ADMA/DDAH pathway plays a crucial role in acute hepatic I/R injury.

### 4.1. Biliary Clearance of ADMA

The liver's ability, as demonstrated in this work, to eliminate ADMA through the bile, supports the previously published results: an increase in plasma ADMA levels is induced only by cirrhosis caused by bile duct ligature (BDL); they are not increased by thioacetamide- (TAA-) induced cirrhosis [20]. Laleman et al. reported that the BDL rats exhibited a decreased rate of ADMA removal of about 50%, probably due to blocked bile excretion, thus inducing an increase in circulating ADMA. The liver necrosis found in BDL rats was lower than that in thioacetamide- (TAA-) induced cirrhosis, thus excluding the involvement of hepatic damage in the accumulation of serum ADMA concentration [20]. In the present work, I/R following a short BDL period induced an increase in intracellular ADMA levels. Moreover, high ADMA plasma concentrations have been found in human alcoholic cirrhosis only in association with high levels of plasma bilirubin [28], suggesting a possible relationship between the compromised biliary excretion of bilirubin and the increase in plasma ADMA. Furthermore, the induction of cirrhosis by BDL caused an increase in plasma levels of both bilirubin and ADMA and not in thioacetamide- (TAA-) induced cirrhosis [20]. The present work reports that, when compared with the sham group, a marked increase in serum bilirubin and significantly higher serum levels of ADMA were found with 60 min ischemia but not 30 min ischemia.

### 4.2. Changes in Tissue, Serum, and Biliary Levels of ADMA after I/R Injury

Rat and human livers have been shown to play an important role in ADMA metabolism by taking up large amounts from the systemic circulation [7]. The present work shows that higher serum levels of ADMA as compared with control livers were found after 60 min ischemia; the same trend was previously reported by Trocha et al. [27]. Plasma ADMA levels are increased in patients with liver cirrhosis [28], alcoholic hepatitis [29], and acute liver failure [13]. In keeping with the increase in serum ADMA levels, we recorded a tissue time-dependent decrease in tissue ADMA content after I/R. Little is known about the changes in tissue ADMA during I/R injury. Interestingly, Cziráki et al. have reported myocardial ADMA during I/R damage, demonstrating that ADMA concentrations are markedly reduced during ischemia and increased during reperfusion [30]. They began the evaluation after 2 hours of reperfusion and this could explain our low ADMA levels observed in liver after 1 hour of reperfusion. Recently, He et al. reported a significant decrease in tissue ADMA levels during acute myocardial infarction [11]. In the present work, after both 30 min and 60 min of ischemia, we recorded a marked increase in biliary ADMA when compared with the respective sham-operated groups. Recently, the plasma concentration of ADMA has been reported to increase significantly until the first postoperative day after cardiopulmonary bypass due to extensive I/R damage, suggesting the use of ADMA as a reliable and feasible marker of early I/R injury [31]. On the other hand, in the 85% of patients who rejected the liver graft, a clear increase in ADMA concentration preceded the onset of the first episode of rejection [32]. Thus, serum ADMA levels, together with biliary ADMA concentrations, could be a potential marker of dysfunction of the liver graft in the posttransplantation period. In particular, the increased levels of biliary ADMA, already detected after 30 min ischemia, preceded the increase in serum levels of ADMA suggesting that the evaluation of ADMA in bile could represent an early marker of liver injury. It is worth noting that previous results demonstrated that plasma ADMA evaluation appears to be an early predictor for survival in patients with sepsis associated with acute liver failure [33].

### 4.3. DDAH and I/R Injury

DDAH is a key regulator of intrahepatic ADMA homeostasis, and to gain more insights into the role of tissue DDAH in the regulation of intracellular, serum, and biliary ADMA levels, the present study evaluates the activity and expression of this enzyme using a rat model of I/R. In particular, DDAH is a cysteine hydrolase enzyme that may be inhibited by oxidative stress [8] and can be suppressed by both superoxide and H_2_O_2_ [34]. To support this hypothesis, we evaluated hepatic MDA and ROS production and the GSH/GSSG ratio. No changes after 30 or 60 min ischemia were found in any of these parameters. As suggested by Hu et al., a more prolonged reperfusion time is needed to obtain a significant increase in MDA levels after hepatic I/R damage [35]. Based on the published results and the present data, this work supports the hypothesis that oxidative stress-independent changes in DDAH activity occur in the liver after acute I/R injury. The decrease in DDAH activity observed after hepatic I/R is associated with a reduction in mRNA and protein expression and an increase in serum ADMA levels. Interestingly, the reduction in DDAH activity, by deleting the gene or by inhibiting its transcription RNAs, has been found to lead to an increase in plasma ADMA levels [36, 37] whereas an overexpression of DDAH in transgenic mice led to a decrease in ADMA levels [38]. Our results support the previous findings that the serum concentration of ADMA is negatively associated with DDAH activity in liver [39]. One possible explanation for the contrasting results obtained by Volti et al. could be connected with reperfusion time: they evaluated the tissue DDAH after 3 hours of reperfusion while we quantified the DDAH activity after only 1 hour of reperfusion [40]. Recent data in rat liver subjected to I/R has demonstrated a decrease in DDAH activity related to the age of rats [17]. We also used young rats and this could justify the decrease of DDAH activity in accordance with Trocha et al. [17]. Moreover, the evaluation of renal I/R injury has demonstrated a reduction in DDAH activity and expression, after 1 hour of reperfusion, leading to an increase in ADMA levels and cardiovascular risks [41]. Thus, we suggest that while intracellular ADMA levels will drop during reperfusion, a concomitant decrease in DDAH activity and expression and a simultaneous ADMA release in the circulation and bile will occur.

DDAH-1 gene regulation in ischemia can be attributed to farnesoid X receptor (FXR). FXR plays critical roles in the transcriptional regulation of various genes. Sequence analysis of the DDAH-1 gene reveals the presence of an FXR response element (FXRE) and a dose-dependent response to FXR antagonist GW4064, in terms of DDAH-1 gene expression, has been shown [42]. Furthermore, FXR suppression has been demonstrated in HEPG2 cells exposed to ischemia. This suppression was p38 MAPK, and not HIF1alpha dependent [43].

DDAH-2 is the predominant isoform of these enzymes in the vasculature as it is found in endothelial cells. Gene silencing of DDAH-2 reduces vascular NO generation and produces vasoconstriction. DDAH-2 is also expressed in the kidney in the* macula densa* and distal nephron [8]. During I/R injury, the main role of degradation of ADMA is conducted by DDAH-1, and the role of DDAH-2 is not so clear. It is known that renin-angiotensin-aldosterone system (RAAS) may promote the progression of I/R injury, especially in the kidney [44]. Furthermore, angiotensin type 1 receptor activation in kidneys reduces the expression of DDAH-1, but increases the expression of DDAH-2. This isoform-specific distribution and regulation of DDAH expression in liver, kidney, and blood vessels provides a potential mechanism for site-specific regulation of NO production [8].

PMRTs involved in the demethylation reaction can be divided into two groups: type I producing ADMA and type II producing SDMA [45]. We evaluated mRNA expression of PMRT-1 at the end of I/R and our results support the recently published data: the highest value was recorded in sham-operated animals not in the I/R group [45].

The kidneys and the liver play a key role in the regulation of the systemic serum concentrations of ADMA. The kidney is mainly involved in urinary excretion and the liver has a major role in ADMA metabolism. In this study, we also found that biliary excretion plays a role in ADMA elimination, and that biliary excretion increases during I/R injury. ADMA can be released in extracellular space by cationic amino acid transporters (CATs) that are also involved in the removal of circulating ADMA by the liver [46]. In the present study, no changes in CAT-2, involved in ADMA transport, were detected after I/R injury. This confirms the recently published data obtained using an ischemic acute kidney injury model [47]. Cellular disposal of ADMA can take place by two mechanisms: degradation by DDAH and export from the cell via CAT [46]. Diminished activity and expression of DDAH-1 and no changes in CAT-2 activity may be involved in the increase in extracellular ADMA levels after I/R.

The hepatoprotective effects of NO have been well documented in several models of liver injury including I/R [18]; thus, the understanding of ADMA/DDAH pathway may be considered as a potential point of interest to reduce the effect of I/R injury.

In conclusion, the main findings of this study are as follows: (1) ADMA is excreted in the bile, as we have documented for the first time, (2) biliary ADMA excretion increases after I/R, and (3) hepatic I/R injury is associated with an oxidative stress-independent alteration in DDAH activity. Although the relationship between the changes in ADMA concentration in serum, bile, and hepatic tissue and hepatic injury needs to be clarified, these data are a fundamental step forward in the development of novel therapeutic strategies that regulate serum, tissue, and biliary levels of ADMA in which the DDAH enzyme plays a crucial role.

## Figures and Tables

**Table 1:** List of forward and reverse primers used in experiments.

Gene	Sequence
Rat DDAH-1	Forward 5′-CAA CGA GGT CCT GAG ATC TTG GC-3′
	Reverse 5′-GCA TCA GTA GAT GGT CCT TGA GC-3′

Rat PRMT-1	Forward 5′-TGC TGC ACG CTC GTG ACA AGT-3′
	Reverse 5′-TCC ACC ACG TCC ACC AGG GG-3′

Rat UBC	Forward 5′-CAC CAA GAA CGT CAA ACA GGA A-3′
	Reverse 3′-AAG ACA CCT CCC CAT CAA ACC-5′

Rat GAPDH	Forward 5′-AAC CTG CCA AGT ATG ATG AC-3′
	Reverse 5′-GGA GTT GCT GTT GAA GTC GTC A-3′

**Table 2:** GSH/GSSG ratio, MDA, and ROS levels after 30 or 60 min ischemia followed by 60 min reperfusion.

	30 min ischemia and 60 min reperfusion		60 min ischemia and 60 min reperfusion	
	Sham	I/R	Sham	I/R
GSH/GSSG	9.1 ± 0.9	8.1 ± 0.5	9.3 ± 1.3	8.3 ± 0.7
MDA (nmol/mg liver)	1.9 ± 0.3	1.8 ± 0.5	2.3 ± 0.4	2.4 ± 0.9
ROS (Arbitrary Unit)	1849 ± 39	1982 ± 71	2019 ± 34	2055 ± 56

The results are reported as the mean ± S.E. of 8 different experiments.

## Abbreviations

ADMA: Asymmetric dimethylarginine
BDL: Bile duct ligature
CATs: Cationic transporters
DDAH: Dimethylarginine dimethylaminohydrolase
PRMT: Protein methyltransferase
SDMA: Symmetric dimethylarginine.
